# Exploring the effects of arch support height on badminton sidestep cutting using musculoskeletal modeling

**DOI:** 10.1371/journal.pone.0341024

**Published:** 2026-01-13

**Authors:** Siqin Shen, Haojie Li, Jin Teng, Xiaowei Wei, Meixi Pan, Fan Yang, Jorge Diaz-Cidoncha Garcia, Qing Yi

**Affiliations:** 1 College of Physical Education, Dalian University, Dalian, Liaoning, China; 2 Guangxi Minzu Normal University, Chongzuo, Guangxi, China; 3 Department of Sports Biomechanics, Beijing Sport University, Beijing, China; 4 Li Ning Sport Science Research Center, Li Ning (China) Sports Goods Company Limited, Beijing, China; 5 Fédération Internationale de Football Association, Zurich, Switzerland; Tokai University School of Medicine, JAPAN

## Abstract

Sidestep cutting is a fundamental yet high-risk movement in badminton, often placing considerable biomechanical stress on the lower limbs. Arch support insoles are commonly used to improve foot stability and redistribute loads, but their specific biomechanical effects during badminton movements remain unclear. This study investigated how different arch support heights influence lower limb mechanics during a 45-degree sidestep cutting (45C) maneuver in badminton players, with a focus on joint kinematics, kinetics, and contact forces. Fifteen male athletes performed 45C maneuver trials under three insole conditions: no support (NS), low support (LS), and high support (HS). Lower limb biomechanics were analyzed using musculoskeletal modeling in OpenSim, assessing joint angles, joint moments, and joint contact forces at the ankle, knee, and hip. A one-way repeated-measures ANOVA was used to evaluate differences across conditions. The high-support condition significantly increased ankle dorsiflexion (P = 0.002) and knee external rotation (P < 0.001) compared with the no-support and low-support conditions. Hip extension moments were higher under the high-support condition (P = 0.043). Anterior–posterior ankle joint contact force increased under high support (P = 0.014), while no significant differences were observed in knee or hip joint contact forces across conditions. These findings describe acute mechanical responses under controlled laboratory conditions. Whether these responses relate to long-term movement strategies in different athlete populations remains to be clarified in future work.

## Introduction

Badminton involves rapid directional changes, explosive movements, and complex footwork patterns. These demands place substantial biomechanical stress on the lower limbs, especially during high-intensity play [1]. Although it is a non-contact sport, badminton has a relatively high incidence of ankle and knee injuries. These injuries are often caused by frequent cutting maneuvers, sudden decelerations, and quick accelerations that repeatedly load the joints [2]. Typical injury types include ligament sprains, muscle strains, and overuse syndromes. When combined with suboptimal biomechanics or poorly fitted footwear, the risk of injury may increase further [2–4].

To reduce these risks, athletes commonly undergo targeted strength and conditioning programs aimed at improving agility, balance, and neuromuscular coordination. However, even well-trained individuals may experience limitations due to anatomical or mechanical characteristics, such as foot arch structure or joint alignment [5–7]. As a result, researchers and practitioners have increasingly explored how external tools, such as footwear modifications, can support movement control and lower injury risk during sports participation [8–11].

Among these interventions, arch support insoles are frequently used to improve foot posture, redistribute plantar pressure, and stabilize the lower limb during dynamic movement. Studies have shown that appropriate arch support can help increase foot-ground contact area and limit excessive pronation, which in turn may lead to more efficient and stable movement patterns [12,13]. In sports that involve lateral movements, such as basketball and baseball, arch support has been associated with improved joint alignment and reduced instability during high-load tasks [14,15]. Similarly, baseball pitchers using arch support insoles have demonstrated enhanced foot and knee stability, which may contribute to better movement efficiency and reduced injury risk [12]. Biomechanically, arch support can help control excessive pronation, which has been linked to increased risk of lower limb injuries, particularly in sports requiring repetitive cutting maneuvers [16]. However, modifying ankle kinematics to enhance local joint stability does not necessarily guarantee improved task performance. In some contexts, increased mechanical constraint at the foot–ankle complex may entail trade-offs between stability, cutting speed, and maneuverability [17]. The present study therefore focused on lower-limb joint mechanics during a standardized 45C maneuver, while potential performance consequences of different arch-support configurations remain an important topic for future work.

At the same time, not all evidence points to clear benefits. Some studies have reported that high levels of arch support may restrict natural foot motion or increase the likelihood of ankle inversion during landing, which could elevate the risk of injury [18]. Differences in study design, sport-specific movement tasks, and participant characteristics may all contribute to these inconsistent findings. Moreover, while research on arch support has been conducted in several sports contexts, relatively little is known about its specific effects in badminton, where unique footwork strategies are used.

One movement in particular, the 45-degree sidestep cutting maneuver (45C), plays a central role in badminton gameplay. This task allows players to reposition quickly and cover the court efficiently. However, it is also associated with high ground reaction forces and increased demands on joint control at the ankle and knee [19]. Although similar movements have been studied in sports such as soccer and basketball, badminton-specific research remains limited. The lighter footwear, faster pace, and distinct court surface in badminton may lead to different biomechanical responses that have not yet been fully explored [20].

A bibliometric search of the Web of Science Core Collection (2000–2024) using the topic query “badminton* AND arch* AND support*” identified only a small number of publications related to arch support or insoles in badminton, and none of these studies directly examined the biomechanical effects of different arch-support heights during the 45C maneuver. This quantitative evidence reinforces that badminton-specific research on arch support remains scarce and highlights the need for targeted biomechanical analyses of sport-specific footwork.

This study aimed to quantify the effects of three arch-support heights on lower-limb kinematics, joint moments, and joint contact forces during the 45C maneuver in competitive badminton players.

We hypothesized that the high-support condition would increase ankle dorsiflexion angle and hip extension moment, and would elevate anterior–posterior ankle joint contact force compared with the no-support and low-support conditions.

## Materials and methods

### Participants

The required sample size was determined using G*Power software for a priori power analysis [21]. The significance level (α) was set at 0.05, with a statistical power (1 – β) of 0.80. A one-way repeated-measures ANOVA was selected as the statistical test, given the single independent variable—arch support height (no support, low support, high support)—evaluated under repeated conditions. The analysis indicated that a minimum of 12 participants was necessary to detect a medium effect size (Cohen's f = 0.25). To ensure robust data collection and account for potential dropouts, 15 male badminton players were prospectively recruited between July 22, 2024, and December 22, 2024. Participants had an average age of 22.67 ± 1.19 years, a height of 1.77 ± 0.02 m, and a body mass of 74.8 ± 7.56 kg. All participants were certified Chinese National Class II badminton athletes competing at the collegiate competitive level, with several years of structured training and tournament experience, which clearly distinguishes them from recreational players. All athletes were predominantly right-leg and right-hand dominant and reported no injuries in the preceding six months. Additionally, none had prior exposure to the specific footwear model used in the study. Foot morphology was screened using a standardized ink-based static footprint test, a commonly used clinical method for identifying abnormal arch patterns [22]. All athletes demonstrated normal medial–longitudinal arches, with no indications of flatfoot or high-arch characteristics. Quantitative measures such as the arch height index or navicular drop were not collected because the footprint screening revealed no clinically meaningful deviations. The study protocol was approved by the university's institutional ethics committee (approval number: GXNU-HEC-2024–001), and written informed consent was obtained from all participants before the study commenced. The participant appearing in Fig 4 provided written informed consent (as outlined in the PLOS consent form) for publication of the identifiable image.

**Fig 1:**
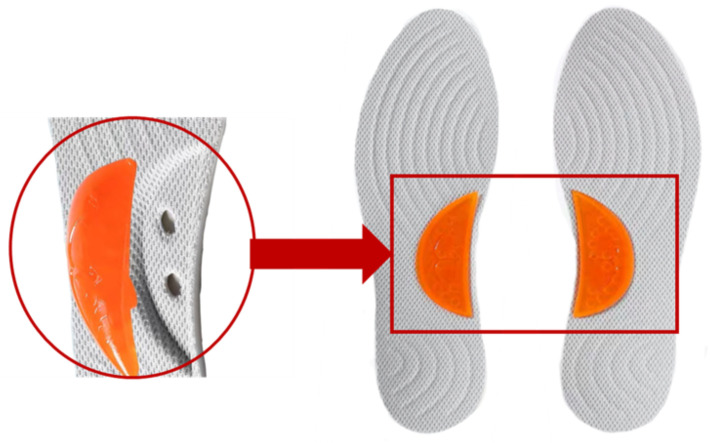
Placement method of the arch support inserts.

**Fig 2:**
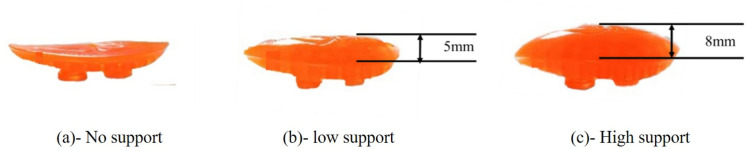
Arch support inserts of varying heights. (a) No support, (b) Low support, (c) High support.

**Fig 3:**
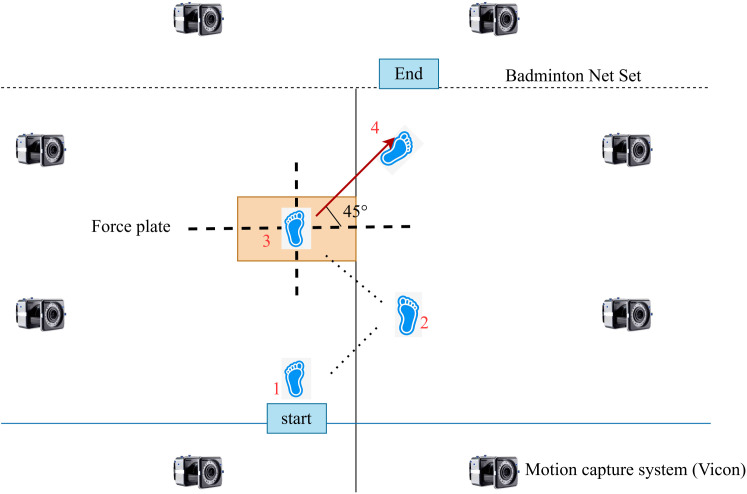
Laboratory trajectory of the 45C maneuver.

### Arch support insoles

This study utilized an insole with a base thickness of 5 mm, constructed from polyurethane (PU). The arch region featured a cut-out design to accommodate interchangeable arch support pads of varying heights, fabricated from thermoplastic rubber (TPR) (Fig 1). Three distinct arch support conditions were evaluated (Fig 2): the no-support (NS) condition consisted of the primary insole without additional elevation, creating a flat profile (Fig 2a); the low-support (LS) condition included a 5 mm elevation above the flat profile (Fig 2b); the high-support (HS) condition incorporated an 8 mm elevation relative to the flat profile (Fig 2c). The 5-mm and 8-mm arch elevations were selected to fall within physiologically reasonable ranges reported for medial arch supports in running footwear and foot orthoses. Previous work has designed arch supports with height “doses” of 0, 4 and 8 mm and demonstrated systematic effects of increasing arch height on plantar pressure distribution and center-of-pressure trajectories during running [23]. In addition, several orthotic intervention studies have used 5-mm wedged or elevated components as a clinically relevant level for modifying lower-limb biomechanics during gait and running [24,25].

To ensure experimental consistency, all participants wore the same standardized badminton shoes (US size 9), featuring identical outsole composition, midsole cushioning properties, and upper material. Additionally, participants wore standardized socks to minimize variations in foot-insole interaction and prevent discrepancies in pressure distribution. The uniform shoe size ensured a snug but comfortable fit, preventing excessive movement inside the shoe and eliminating potential confounding effects related to footwear variability.

### Movement tasks

The 45C maneuver was selected as the test movement due to its strategic significance in badminton footwork. Although not the most frequently employed movement, 45C maneuver is crucial for rapid directional changes, enabling tactical advantages during gameplay. This maneuver demands precise force redistribution, agility, and dynamic postural control, making it an appropriate task for assessing the biomechanical effects of arch support variations [26]. Given the injury risks associated with lateral cutting, analyzing this motion provides valuable insights into how different insole conditions influence lower limb biomechanics [27].

Participants performed five successful trials per insole condition, leading to a total of 15 trials per participant. The movement speed during the approach phase was self-selected to reflect each athlete's natural cutting strategy. Before formal data collection, participants completed several familiarization trials to ensure consistent task execution.

A trial was considered successful if the left foot made full contact with the force plate, the sidestep cut at a 45° angle was executed as instructed, and no observable execution errors occurred. A visual target (10 cm × 10 cm) was taped onto the central region of the force plate to guide foot placement. Trials were accepted only when the center of pressure at initial contact fell within 5 cm of the target center, and trials outside this range were discarded and repeated. The standardized protocol was as follows: participants moved forward, allowing their left foot to contact the target region on the force plate; upon left foot ground contact, they performed a 45° sidestep cut to the right, pushing off forcefully with the left foot to initiate the cutting motion; they then decelerated along the cutting trajectory and stabilized their movement, ensuring controlled stopping and postural balance (Fig 3). This protocol ensured consistency across trials and allowed for a detailed assessment of how different arch support heights influenced lower-limb mechanics during rapid directional changes.

### Experimental equipment and protocol

**Fig 4:**
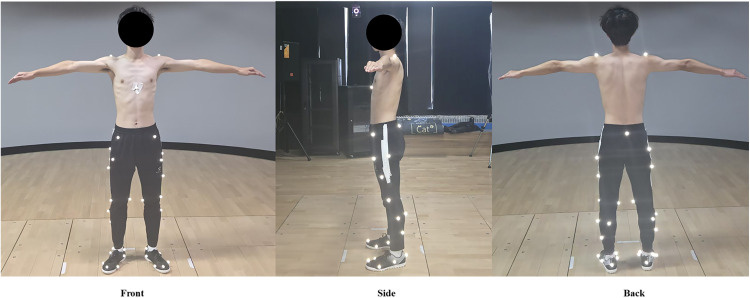
Anterior, lateral, and posterior views of a participant with spherical reflective markers and surface EMG electrodes placed according to the standard Gait 2392 marker protocol. Reflective markers were positioned over the anatomical landmarks required for model scaling and inverse kinematics, and EMG electrodes were attached following SENIAM guidelines. The participant provided written informed consent for publication of the identifiable images.

Kinematic data were collected using an 8-camera Vicon motion capture system (Vicon Motion Systems Ltd., Oxford, UK) sampling at 200 Hz. Ground reaction forces were recorded at 1,000 Hz using two embedded force platforms (Kistler Instrument AG, Winterthur, Switzerland). All devices were synchronized within the Vicon Nexus acquisition environment to ensure frame-level alignment across modalities. Marker placement followed the OpenSim Gait 2392 convention, as illustrated in Fig 4.

Muscle activity was recorded at 1,000 Hz using the Trigno wireless surface electromyography (EMG) system (Delsys, Boston, MA, USA). Ten lower-limb muscles were monitored bilaterally: rectus femoris, biceps femoris, tibialis anterior, medial gastrocnemius, and lateral gastrocnemius. Although the biomechanical analysis focused on the supporting leg, bilateral EMG was collected to ensure reliable timing identification and consistent synchronization during model validation. Electrode placement followed SENIAM-based procedures.

Before testing, a static calibration trial was captured for model scaling. Participants completed a self-selected 5-minute warm-up to familiarize themselves with the laboratory layout. The sidestep cutting maneuver was performed along a 9-m approach runway toward a force-plate-embedded landing zone. Tight-fitting athletic attire was worn to reduce soft-tissue artifacts.

Participants performed tasks under three insole conditions (NS, LS, HS). All trials were performed with the same shoe model to control for torsional stiffness. Five successful trials were collected per condition (15 total). A one-minute rest was provided between trials and a ten-minute rest between insole conditions. Condition order was randomized to minimize order effects.

### Musculoskeletal model

A generic OpenSim Gait 2392 model was used, comprising 10 rigid bodies, 23 degrees of freedom, and 92 musculotendon units [28]. Kinematic data and ground reaction forces were processed and converted into TRC and MOT files using MATLAB R2022b (MathWorks, Natick, MA, USA). Data processing adhered to the standardized workflow in OpenSim v4.3 [29]. No additional markers or sport-specific constraints were introduced; the standard marker set was sufficient to capture the range of motion required for the sidestep cutting task.

The model was scaled to match each participant's anthropometric data, derived from static marker positions and body weight. All segment dimensions were adjusted individually, and the existing joint definitions in Gait2392 were retained. Scaling accuracy was validated by ensuring that the root mean square error (RMSE) between experimental and virtual marker positions was less than 0.02 m, with a maximum error not exceeding 0.04 m.

In OpenSim, joint kinematics were computed using inverse kinematics, which minimizes the weighted squared error between experimental and model marker positions. Joint kinetics were subsequently obtained through inverse dynamics by combining the kinematic solution with segmental inertial properties and ground reaction forces. This workflow allowed the standard Gait 2392 marker set to provide sufficient information for resolving three-dimensional lower-limb joint motions during the sidestep cutting maneuver.

Joint moments were calculated through inverse dynamics, followed by static optimization, which estimated muscle activations and forces by minimizing the sum of squared muscle activations. The calculated muscle forces were then used to estimate joint contact forces.

### Data and statistical analysis

Prior to testing, maximal voluntary contraction (MVC) of the muscles was assessed using isometric strength evaluations. Raw EMG signals were band-pass filtered (100–500 Hz) using a fourth-order Butterworth filter and then rectified before RMS computation. Amplitude analysis was performed using root mean square (RMS) calculations to derive MVC and normalized activity values for each motion. Muscle activation levels were expressed as the ratio of the RMS amplitude of each motion to the RMS amplitude of the MVC, ranging from 0 (no activation) to 1 (full activation). Experimental muscle activation levels were compared with those estimated via static optimization to validate the musculoskeletal model.

Biomechanical data were analyzed specifically from the stance phase of each test action. Stance phase was defined from initial ground contact (identified as the first frame in which the vertical ground reaction force exceeded 20 N) to toe-off (the frame at which the vertical ground reaction force returned below 20 N). For interpretability, the stance phase was further divided into braking and propulsion subphases, with the transition point defined at the instant of peak knee flexion, a commonly used criterion in cutting-movement analysis. Peak joint angles, joint moments, and joint contact forces were identified from the entire stance phase. This interval includes the braking period in which peak joint loading typically occurs during a sidestep cut, and therefore adequately captures the injury-relevant portion of the movement. All trials were visually inspected to ensure that only the support foot contacted the force plate. Trials showing any non–support-foot contact or brief double-limb stance at either the entry or exit of the task were discarded and recollected to maintain data validity. Joint angles, joint moments (normalized to body weight: Nm/kg), and joint contact forces (expressed as %BW) were extracted from this phase for statistical analysis. Statistical analyses were performed using SPSS 27.0 (IBM Corp., Armonk, NY, USA). A one-way repeated-measures ANOVA assessed the effects of arch support height (P < 0.05), with post-hoc Bonferroni adjustments applied for pairwise comparisons (NS, LS, HS). Because all participants completed all three insole conditions, this model accounted for the within-subject dependence of observations.

Given the biomechanical coupling among joint angles, joint moments, and joint contact forces, and because ankle dorsiflexion, knee external rotation, hip extension moment, and ankle anterior–posterior contact force were defined a priori as primary outcomes, no global correction across all dependent variables was performed. The remaining variables were treated as secondary exploratory measures.

### Ethics statement

The study protocol was reviewed and approved by the Medical Ethics Committee of Guangxi Minzu Normal University (Approval Number: GXNU-HEC-2024–001). All procedures complied with the Declaration of Helsinki and relevant national ethical guidelines. Written informed consent was obtained from all participants. The individual appearing in Fig 4 has given written informed consent (as outlined in the PLOS consent form) to publish the identifiable image.

## Result

### Model validation

Static optimization was adopted because it provides a stable and widely implemented approach for estimating muscle forces and joint loading in rapid cutting tasks. Given that the primary outcomes of this study were joint moments and joint contact forces, static optimization offered a well-validated framework with computational robustness and consistency across participants. EMG data were used qualitatively to verify activation timing rather than to drive the optimization, as reproducing EMG amplitudes was not the aim of the analysis. Although only the supporting leg was analyzed, EMG was recorded bilaterally to ensure consistent identification of timing events and to improve robustness during model validation.

Five superficial lower-limb muscles were selected for validation (tibialis anterior, medial gastrocnemius, lateral gastrocnemius, rectus femoris, and biceps femoris), as these muscles provide reliable surface EMG signals with minimal cross-talk during rapid cutting movements and represent key contributors to ankle and knee control during the 45C maneuver. Deep muscles such as the soleus or semitendinosus were not included because surface EMG cannot capture their activation patterns with sufficient accuracy during high-speed dynamic tasks [30,31].

**Fig 5:**
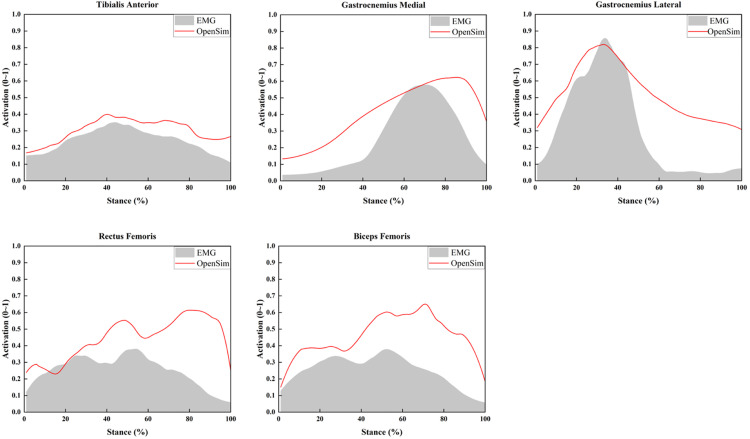
Comparison of tibialis anterior, medial gastrocnemius, lateral gastrocnemius, rectus femoris and biceps femoris muscle activation level obtained by EMG signal and OpenSim optimization algorithm.

Fig 5 illustrates the comparison between static optimization -predicted muscle activations and experimentally recorded EMG envelopes during the 45C maneuver. The agreement in activation timing and the similarity of overall profile characteristics support the reliability of the musculoskeletal model for representing lower-limb muscle activity during the task. Because surface EMG envelopes and model-derived activation estimates differ in their physiological basis, correlation coefficients were not calculated; validation focused instead on the temporal alignment and qualitative pattern consistency.

Although the OpenSim model generates activation estimates for all lower-limb muscles, including deep shank muscles such as the tibialis posterior, only the superficial muscles with reliable surface EMG signals were included in the validation and analysis.

### Joint angles

**Fig 6:**
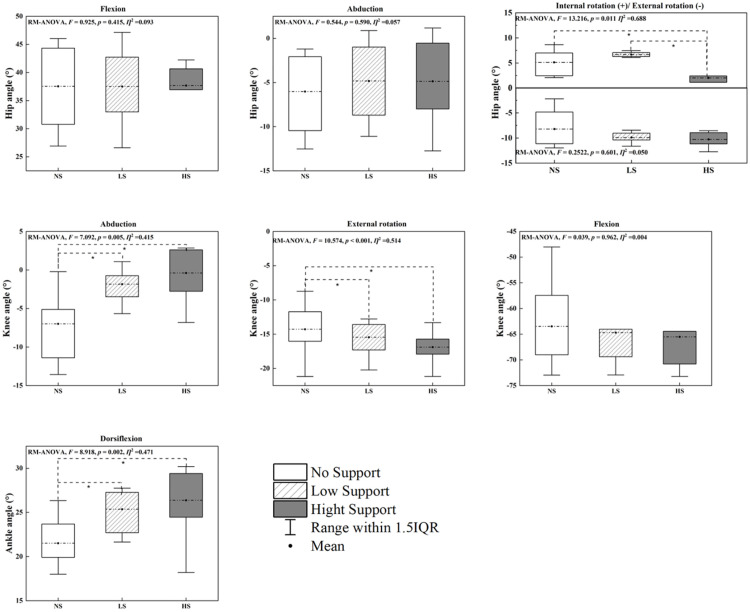
Peak joint angles for the ankle, knee, and hip across three insole conditions (NS, LS and HS). Source data and complete descriptive statistics for this figure are provided in S1 Data. * Indicates significant differences between the insole conditions (P < 0.05).

The repeated-measures ANOVA (RM-ANOVA) identified significant effects of arch support height on lower limb joint angles (Fig 6). At the hip joint, internal rotation was significantly influenced by insole conditions (P = 0.011), with the HS condition producing greater rotation compared to both NS and LS conditions. In contrast, no significant differences were observed for hip flexion (P = 0.415) or abduction (P = 0.590) across the insole variations.

At the knee joint, significant main effects were detected for external rotation (P < 0.001) and abduction (P = 0.008). Post-hoc analyses revealed that the HS condition resulted in significantly greater external rotation and abduction angles compared to NS. However, knee flexion did not exhibit significant differences across the insole conditions (P = 0.862).

For the ankle joint, dorsiflexion angles varied significantly across insole conditions (P = 0.002). Post-hoc analyses indicated that the HS condition led to significantly greater dorsiflexion compared to both NS and LS, highlighting enhanced ankle mobility under high-support conditions. These findings indicate that arch support height primarily influences ankle dorsiflexion, whereas knee adaptations reflect changes in rotational alignment rather than increases in joint mobility.

### Joint moments

**Fig 7:**
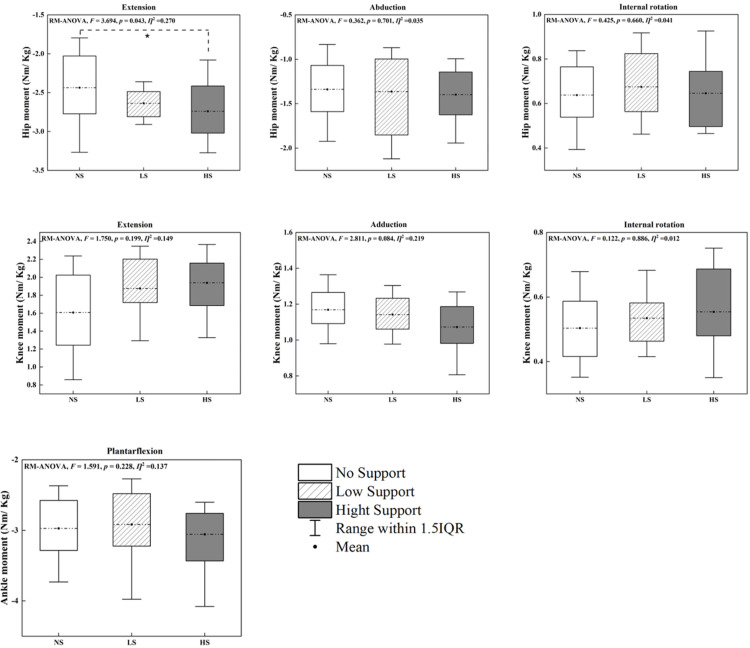
Peak joint moments for the ankle, knee, and hip across three insole conditions (NS, LS and HS). Source data and complete descriptive statistics for this figure are provided in S1 Data. * Indicates significant differences between the insole conditions (P < 0.05).

The effects of arch support height on joint moments were primarily observed at the hip joint, reflecting its pivotal role in lower limb biomechanics (Fig 7). Hip extension moments were significantly influenced by insole conditions (P = 0.043), with the HS condition generating higher moments compared to NS. However, no significant differences were observed for hip abduction (P = 0.701) or internal rotation (P = 0.660) moments across the insole conditions.

At the knee joint, none of the evaluated moments, including extension (P = 0.199), adduction, or internal rotation, exhibited significant differences among the insole conditions. Similarly, ankle joint plantarflexion moments did not demonstrate significant effects (P = 0.228). The limited effects observed at the knee and ankle joints suggest that arch support variations predominantly impact proximal joints, such as the hip, during movements requiring powerful propulsion and stabilization, as seen in badminton.

### Joint forces

**Fig 8:**
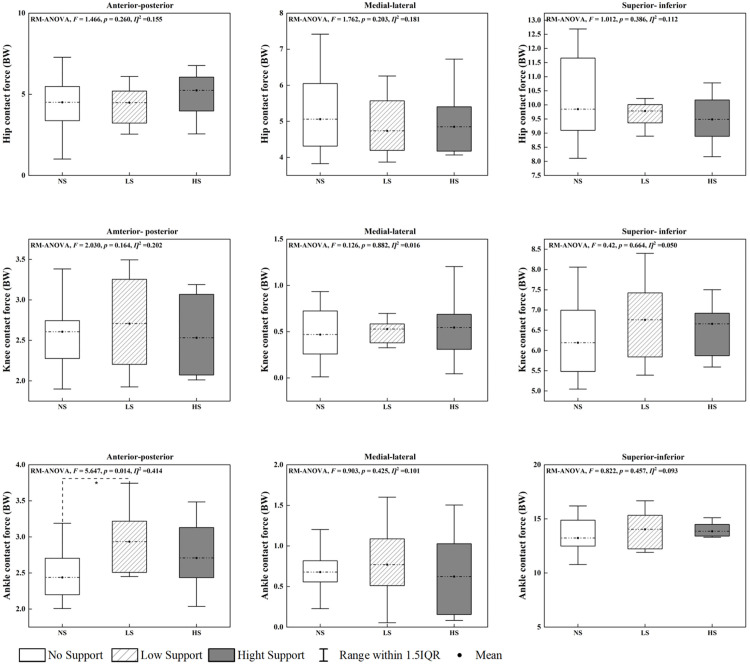
Peak joint contact forces for the ankle, knee, and hip across three insole conditions (NS, LS and HS). Source data and complete descriptive statistics for this figure are provided in S1 Data. * Indicates significant differences between the insole conditions (P < 0.05).

Significant effects of arch support height on joint contact forces were observed at the ankle joint, particularly in the anterior-posterior direction (Fig 8). Anterior-posterior forces exhibited a significant main effect (P = 0.014), with the HS condition producing significantly greater forces compared to NS. However, medial-lateral (P = 0.425) and superior-inferior (P = 0.457) forces did not differ significantly across the insole conditions.

At the knee joint, no significant differences were detected for anterior-posterior (P = 0.164), medial-lateral (P = 0.882), or superior-inferior (P = 0.664) forces. Similarly, no significant effects were observed at the hip joint across all force directions (P > 0.05). The increase in anterior-posterior forces at the ankle under the HS condition suggests enhanced load absorption and force distribution, which are critical for the high-impact and lateral movements characteristic of badminton.

## Discussion

The present study quantified how different arch-support heights influence lower-limb mechanics during the badminton 45C maneuver, revealing several novel biomechanical responses beyond the general role of insoles in lateral cutting. Although arch support modulated foot–ankle kinematics, knee and ankle joint moments remained unchanged across conditions, indicating that proximal joint loading was largely preserved despite altered foot mechanics. Conversely, the high-support condition increased anterior–posterior ankle joint contact force, suggesting that elevated arch structures may redistribute loading toward shear components during rapid directional changes. These findings refine current understanding of how arch-support height influences multi-joint coordination in badminton-specific cutting.

Mechanistically, increasing arch-support height elevates the medial longitudinal arch and reduces midfoot deformation during loading. This structural modification constrains subtalar joint eversion and limits excessive pronation, which shifts the talocrural joint toward a more dorsiflexed orientation [32,33]. Because subtalar motion is coupled with tibial rotation, reduced pronation results in an external rotation bias of the tibia, producing the knee external rotation observed in the high-support condition [34,35]. These distal-to-proximal adjustments explain why ankle dorsiflexion and knee rotation were affected, whereas knee and hip joint moments remained unchanged, consistent with the notion that foot orthoses primarily act by modifying foot–ankle alignment and lower-limb coupling rather than by altering overall neuromuscular output [36].

These findings underscore the biomechanical implications of arch support customization while emphasizing that mechanical changes do not directly indicate performance or injury-related benefits in badminton footwear, particularly for athletes engaged in high-impact, multidirectional movements. Further exploration of joint kinematics, kinetics, and contact forces provides a more detailed understanding of how arch support influences badminton-specific biomechanics.

Our findings indicate that variations in arch support height significantly influence joint kinematics, particularly at the ankle and knee joints. Ankle dorsiflexion angles increased under HS conditions, indicating that greater arch support may alter push-off mechanics and foot–ground interaction patterns during lateral movements [37]. This adaptation may be a response to improved plantar load distribution, without implying improvements in performance or propulsion efficiency, which is essential for deceleration and stability during sidestep cutting. Additionally, higher arch support may reduce excessive pronation in a mechanical sense, although its relevance to actual ankle‑sprain risk cannot be inferred from the present data [38]. In the present study, the high-support insole increased ankle dorsiflexion by approximately 5°—about a 24% increase relative to the no-support condition. Changes of this magnitude fall within ranges associated with altered loading patterns in previous work. The high-support condition also showed a modest decrease in medio-lateral ankle joint contact force, indicative of reduced frontal-plane ankle loading. Such reductions align with evidence linking adequate dorsiflexion to more favorable joint-loading patterns and improved stability throughout the kinetic chain, although any implications for inversion‑related injury risk remain uncertain [39]. Although the absolute changes are moderate, these results highlight that even modest adjustments in arch support and resulting dorsiflexion can produce biomechanically meaningful effects in badminton-specific cutting tasks.

At the knee joint, greater arch support was associated with increased external rotation and abduction angles, which likely represent compensatory adjustments to manage segmental alignment and load distribution rather than indicating functional advantages from the ground [40]. Increased knee abduction may indicate altered tibiofemoral joint loading, which could have implications for anterior cruciate ligament (ACL) strain. Previous studies suggest that foot orthoses can influence knee kinematics indirectly by modifying ankle movement patterns, particularly in high-demand, multidirectional sports [41]. Our findings align with this perspective, highlighting how changes at the foot-ankle complex propagate upward, necessitating coordinated movement adaptations at the knee. Importantly, although knee external rotation increased under the high-support condition, neither knee abduction angle nor knee abduction moment changed significantly, and anterior–posterior knee joint contact forces also remained unchanged. Therefore, the observed kinematic adaptations do not reflect loading patterns typically associated with increased ACL injury risk [42].

At the hip joint, internal rotation increased significantly under HS conditions, further reinforcing the interconnected nature of the lower limb kinetic chain. This kinematic shift may be influenced by modifications in plantar load distribution, where greater arch support alters foot-ground contact mechanics, resulting in upstream biomechanical adjustments [40]. Increased hip internal rotation may enhance kinetic chain continuity, which may reflect upstream adjustments in kinetic-chain coordination rather than improved force transmission, which is crucial for generating explosive movements and maintaining movement efficiency in badminton.

Overall, these findings suggest that arch support influences joint kinematics beyond localized foot adjustments, extending to the knee and hip. While these modifications may modify propulsion-related mechanics and movement control, their implications for long-term neuromuscular adaptations and injury susceptibility warrant further investigation. Future research should explore how individualized arch support configurations influence movement efficiency over time, considering factors such as athletic skill level, sex-specific biomechanics, and chronic adaptations to footwear modifications.

Analysis of joint moments revealed that high arch support significantly influenced hip joint kinetics, with hip extension moments being significantly higher under HS conditions compared to no support (NS) (P = 0.043). This suggests that increased arch support modifies posterior‑chain loading patterns without indicating enhanced propulsion or energy transfer in rapid directional changes [43]. The ability to generate greater hip extension moments under HS conditions may redistribute mechanical demand toward the hip, although implications for propulsion efficiency or knee‑injury risk cannot be determined from the present results in badminton [44].

In the musculoskeletal simulations, the activation estimates of the superficial muscles showed a pattern consistent with the joint-level findings. Hip extensor muscles (particularly the gluteus maximus and hamstrings, as represented in the Gait2392 model) exhibited slightly higher activation under the high-support condition, aligning with the increased hip-extension moment. In contrast, ankle plantarflexor and knee extensor activations remained relatively stable across conditions, mirroring the lack of significant differences in ankle and knee joint moments. Although deep shank muscles cannot be validated with surface EMG, the simulated profiles did not show systematic condition-dependent changes that would alter the interpretation of joint-level mechanics.

A plausible mechanism is that increasing arch-support height alters plantar pressure distribution by shifting load toward the medial arch and forefoot and reducing midfoot deformation during the initial braking phase [45,46]. These changes place the ankle in a more dorsiflexed and mechanically rigid alignment, increasing the demand on the posterior chain to generate sagittal-plane propulsion. Consistent with kinetic-chain concepts, footwear and injury-model studies have shown that when distal joint mechanics are constrained or modified, joint loading and neuromuscular control tend to shift proximally, with greater contributions from the hip extensors during landing and change-of-direction tasks [47]. This distal-to-proximal redistribution of mechanical demand provides a biomechanically plausible explanation for the elevated hip-extension moment observed under the high-support condition in the present study.

The biomechanical mechanisms underlying these adaptations may be linked to altered neuromuscular control and modified ground reaction force distribution. High arch support has been suggested to influence the activation patterns of the hip extensor muscles, particularly the gluteus maximus and hamstrings, which play a key role in stabilizing the pelvis and facilitating powerful push-offs during lateral movements [48]. Increased reliance on hip extension could redistribute the mechanical workload away from the knee joint, potentially lowering the incidence of patellofemoral stress injuries [49].

However, no significant differences in knee or ankle joint moments were observed across insole conditions, suggesting that the influence of arch support on joint kinetics remains localized primarily to the hip joint. This finding aligns with previous studies indicating that proximal joint adaptations play a critical role in mediating energy transfer during dynamic sporting maneuvers. Specifically, the hip serves as a primary force generator, while the knee and ankle function more as stabilizers during lateral cutting tasks in badminton [20].

Joint contact force analysis revealed that high arch support insoles significantly increased anterior-posterior contact forces at the ankle joint (P = 0.014), suggesting that HS insoles alter the pattern of load absorption and redistribution during dynamic movements [50]. This effect is particularly relevant in badminton, where athletes frequently execute forceful push-offs and rapid lateral accelerations, demanding optimized foot-ground interactions for effective force transmission [51].

The observed increase in anterior-posterior forces at the ankle may stem from the elevated arch support modifying the foot's contact dynamics with the ground, shifting the load distribution anteriorly. This redistribution modifies push‑off mechanics but could also increase localized stress on the ankle structures, necessitating further exploration of long-term effects [52].

At the knee and hip joints, no significant differences in joint contact forces were observed across insole conditions (P > 0.05), suggesting that arch support predominantly affects the foot-ankle complex rather than inducing substantial kinetic alterations at proximal joints [53]. These findings align with previous research indicating that foot orthoses modulate local biomechanics without drastically altering global kinetic patterns, reinforcing the need for sport-specific interventions [54]. This lack of proximal joint changes is biomechanically plausible, as knee and hip joint contact forces are largely driven by the magnitude and timing of the vertical ground reaction force and the corresponding sagittal-plane extensor moments, both of which remained relatively consistent across insole conditions in our protocol. In contrast, changes in arch support height primarily influenced foot posture and ankle alignment—mechanical adjustments that tend to redistribute load locally within the distal segment rather than altering whole-body center-of-mass dynamics. Similar distal-dominant responses have been reported in studies examining the effects of foot orthoses and arch-support insoles, where substantial adaptations at the ankle coexist with minimal changes in knee or hip loading [40,55,56]. From a biomechanical perspective, this pattern indicates that modifying arch support height can alter ankle mechanics, although no conclusions regarding injury prevention can be drawn without imposing additional compressive loading on the knee or hip, joints that already experience high cumulative loads during badminton play.

The absence of significant knee and hip force adaptations highlights the specificity of arch support effects, emphasizing that while insoles can optimize force management at the foot-ankle interface, they do not necessarily alter mechanical demands at higher joints. This underscores the importance of customized footwear strategies, particularly for high-intensity multidirectional sports, where controlled force redistribution may influence overuse‑related loading patterns, but effects on injury risk or movement efficiency remain speculative [53]. Although arch-support height produced measurable changes in joint kinematics and contact forces, these findings represent acute mechanical responses observed under controlled laboratory conditions. They should not be interpreted as direct evidence of performance enhancement or injury prevention in real-world badminton play. Arch support is not a comprehensive solution for ankle injuries, and whether the observed mechanical adaptations translate into meaningful functional or clinical outcomes requires confirmation through prospective and longitudinal research.

Future innovations in badminton footwear should explore dynamic arch support systems that adapt to varying movement intensities. Such advancements could incorporate adaptive cushioning materials or smart insoles that provide real-time biomechanical adjustments, further optimizing movement efficiency and reducing injury risk [57]. In this context, our findings may help to inform the design space for emerging adaptive arch-support technologies. Recent developments in 3D-printed customized arch-support insoles allow clinicians to fine-tune arch height, medial posting, and stiffness distribution to the individual's foot morphology and functional demands, and have been shown to improve gait mechanics and ankle alignment in people with functional flat foot and other foot-related musculoskeletal conditions [58–60]. Such adjustable or customizable platforms provide a practical means to translate the present results into sport-specific applications, for example by targeting arch support configurations that enhance ankle dorsiflexion and frontal-plane stability during cutting tasks while avoiding excessive loading at more proximal joints. From a development perspective, these systems should be evaluated against predefined, quantifiable biomechanical targets, such as increasing ankle dorsiflexion range of motion and improving landing or cutting postures that have been linked to lower injury risk [61–63], and avoiding ankle inversion velocities approaching thresholds proposed for sprain identification during sport-specific tasks [64]. The ankle joint contact force profiles reported in this study represent laboratory‑based mechanical responses, particularly in the anterior–posterior direction, provide baseline reference magnitudes that future smart insole or adaptive arch-support prototypes could aim to maintain or reduce during high-intensity cutting maneuvers.

While this study provides valuable insights into the biomechanical effects of arch support on badminton-specific movements, several limitations should be considered. The study was conducted on a relatively small sample of collegiate badminton players, which may limit the generalizability of the findings to professional or recreational athletes. Additionally, the controlled laboratory setting may not fully replicate real-game conditions, where factors such as fatigue, psychological stress, and variable playing surfaces can influence biomechanics. Furthermore, this study focused on acute biomechanical responses; future longitudinal research is needed to examine the long-term effects of arch support on injury prevention and performance optimization. Lastly, gender-based biomechanical differences were not explored, warranting future studies to assess whether arch support interventions yield similar effects in male and female athletes. Although the present study focused on joint-level biomechanics, future research may extend these findings by examining muscle activation or muscle-force responses under different arch-support configurations. In addition, the musculoskeletal simulations were based on the generic OpenSim Gait 2392 model, which was originally developed for gait analysis and may not fully capture the joint mechanics specific to rapid cutting movements. The static optimization algorithm also estimates muscle forces using a simplified cost function that may not entirely reflect the neuromuscular strategies used during explosive changes of direction. These modeling assumptions may influence absolute joint moment and contact force magnitudes, although the relative differences among arch-support conditions remain interpretable under consistent modeling settings.

## Conclusion

This study showed that increasing arch-support height altered lower-limb biomechanics during the badminton 45C maneuver, characterized by greater ankle dorsiflexion and higher anterior–posterior ankle joint contact forces, while knee and hip kinetics remained largely unchanged. These results highlight the localized mechanical influence of arch-support height at the foot–ankle complex and provide baseline biomechanical reference values relevant for understanding badminton-specific footwear mechanics. The present findings describe acute mechanical adaptations under controlled laboratory conditions; whether these responses translate to long-term changes in movement strategy or athlete-specific performance remains uncertain. Future studies should examine chronic adaptations, inter-individual variability, and sport-specific contextual factors to better understand how different arch-support configurations influence movement control in real-game environments.

## Supporting information

S1 Data Minimal dataset containing metadata, joint angle waveforms, joint moment waveforms, joint contact force waveforms, and values used to generate the figures in the manuscript. (XLSX)
